# Transcriptomic Analysis Reveals Molecular Mechanisms of *Wolbachia*–Plant Association

**DOI:** 10.3390/ijms27093746

**Published:** 2026-04-23

**Authors:** Qiancheng Wei, Xinlei Wang, Kedi Zhao, Sha Wang, Ali Basit, Feng Liu, Yiying Zhao

**Affiliations:** College of Agriculture, Shihezi University, Shihezi 832003, China; 13002637216@163.com (Q.W.); 20222012077@stu.shzu.edu.cn (X.W.); zhaokedibs@163.com (K.Z.); 20222012067@stu.shzu.edu.cn (S.W.); basitali27297@stu.shzu.edu.cn (A.B.); liufeng@shzu.edu.cn (F.L.)

**Keywords:** spider mite, endosymbiont, *Wolbachia*, transcriptomics, photosynthesis

## Abstract

Endosymbiotic bacteria in insects are known to influence plant–insect interactions by altering host plant physiology. This study reveals that the endosymbiont *Wolbachia* significantly impairs photosynthesis in cotton plants. Comparative transcriptomic analysis of cotton leaves infested by *Wolbachia*-infected spider mites (Tt-I) and uninfected spider mites (Tt-UI) identified 1912 differentially expressed genes (DEGs). Photosynthesis was the most adversely affected biological process, with 17 genes downregulated in the photosynthesis pathway (e.g., key genes psbW and PETF), as supported by GO and KEGG enrichment analyses. Gene co-expression network analysis further highlighted core genes involved in photosynthesis disruption and carbon fixation. Physiological assessments showed that *Wolbachia* infection led to significantly reduced chlorophyll content and elevated reactive oxygen species (ROS) levels, inducing oxidative stress. These findings demonstrate that *Wolbachia* disrupts cotton photosynthesis through transcriptional repression and ROS-mediated oxidative stress, providing novel insights into plant–insect-symbiont interactions and a theoretical basis for managing mite pests in cotton.

## 1. Introduction

Plant–herbivore interactions are complex and dynamic, representing a longstanding focus in ecology and evolutionary biology [1]. Recent research has increasingly highlighted the pivotal role of insect symbionts in modulating these interactions [2]. Among these symbionts, *Wolbachia*, a widespread intracellular bacterium in arthropods, is renowned for manipulating host reproduction [3,4]. Critically, *Wolbachia* can also alter host physiology, such as by modifying feeding behavior and nutritional metabolism, thereby indirectly influencing plant physiological responses [5,6]. A notable example is the induction of “green islands” on apple leaves by *Wolbachia*-infected leaf miner (*Phyllonorycter blancardella*), where cytokinin levels are manipulated to maintain nutrient-rich tissues [7]. Similarly, symbiotic bacteria in other herbivores, like the Colorado potato beetle, can suppress plant defenses through oral secretions [8]. These findings underscore that the plant–herbivore interface is often a tripartite system, where microbial partners are key players.

The spider mite *Tetranychus turkestani*, a major pest of cotton in Xinjiang, harbors *Wolbachia* infections that significantly impact its own reproduction and detoxification metabolism [9,10]. However, the potential to influence cotton physiology, particularly the foundational process of photosynthesis, remains largely unexplored. Photosynthesis is paramount for cotton yield and quality [11,12,13], and mite feeding damages leaf structures, potentially impairing this vital function [14]. Furthermore, plant responses to biotic stress involve complex physiological shifts, including amino acid metabolism [15], chlorophyll content dynamics [16], and reactive oxygen species (ROS) signaling, which can have dual roles in defense and damage [17,18,19].

Despite these insights, a critical knowledge gap exists regarding the molecular and physiological mechanisms by which *Wolbachia* infection in spider mites affects cotton. It is unknown whether *Wolbachia* exacerbates the suppression of cotton photosynthesis and, if so, whether this occurs through transcriptional reprogramming and/or the induction of oxidative stress.

This study aims to bridge this gap by systematically investigating the impact of *Wolbachia*-infected *T. turkestani* on cotton. We hypothesize that *Wolbachia* infection in spider mites disrupts cotton photosynthesis via transcriptional and oxidative stress pathways. Using an integrated approach combining transcriptomics with physiological analyses, we assessed gene expression changes, amino acid profiles, chlorophyll content, and ROS levels in cotton leaves infested with either *Wolbachia*-infected (Tt-I) or uninfected (Tt-UI) mites. Our findings provide novel mechanistic insights into the *Wolbachia*–plant association and advance the understanding of multitrophic interactions in agro-ecosystems.

## 2. Results

### 2.1. RNA Transcriptome Sequencing Analysis

**Figure 1:**
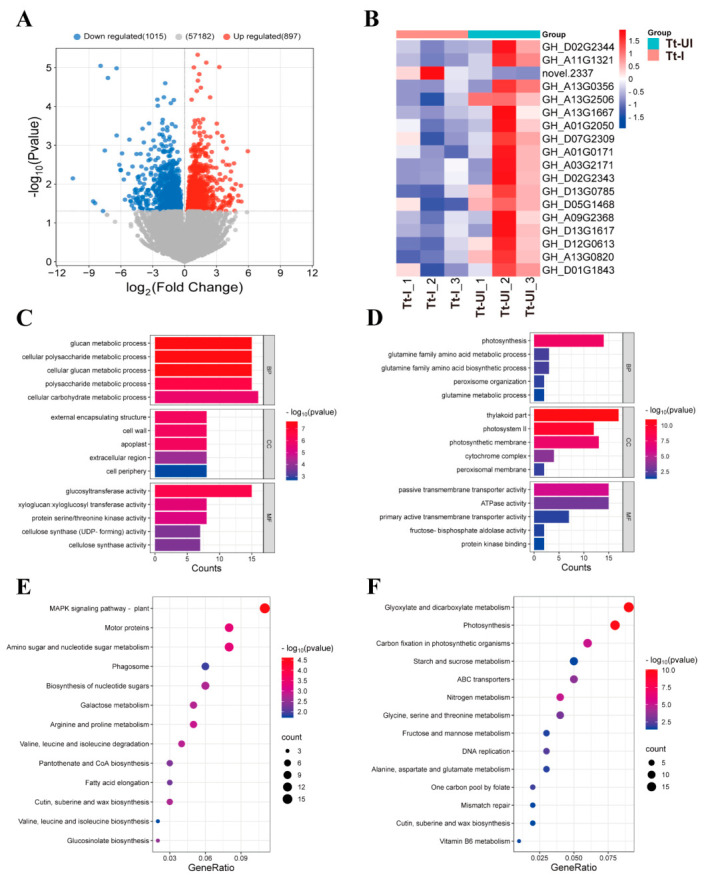
(**A**) Volcano plot of differentially expressed genes (DEGs) between Tt-I and Tt-UI; (**B**) clustering heatmap of important DEGs in photosynthesis; (**C**) GO functional classification of upregulated genes; (**D**) GO functional classification of downregulated genes; (**E**) KEGG enrichment bubble plot of upregulated expression; and (**F**) KEGG enrichment bubble plot of downregulated expression. Tt-UI: Uninfected *Wolbachia* spider mite-infected leaves; Tt-I: *Wolbachia*-infected spider mite-infected leaves.

To compare the plant responses, we performed RNA-Seq analysis on cotton leaves following infestation by either *Wolbachia*-infected (Tt-I) or *Wolbachia*-uninfected (Tt-UI) spider mites. The Q20 values of all samples were above 98%, and the Q30 values were above 95% (sequencing error rate < 1%). GC content was over 43% for all samples, and 92.65–93.63% of unique reads were mapped to the cotton (*Gossypium hirsutum*) genome (Supplementary Table S1). A total of 1912 differentially expressed genes (DEGs) were identified between Tt-I and Tt-UI, including 897 upregulated and 1015 downregulated genes (Figure 1A, Supplementary Figure S1).

GO and KEGG enrichment analyses were performed on DEGs in Tt-I vs. Tt-UI. In GO enrichment (*p* < 0.05), the most enriched terms were functionally classified into biological processes, cellular components, and molecular functions. In biological processes category, photosynthesis was the most enriched and downregulated; In cellular component category, thylakoid part, photosystem II and photosynthetic membrane were the most enriched and downregulated; In molecular function category, passive transmembrane transporter activity and ATPase activity were the most enriched and downregulated (Figure 1C,D). KEGG pathway enrichment identified six significantly enriched pathways (*p* < 0.01), including photosynthesis, glyoxylate and dicarboxylate metabolism, nitrogen metabolism, cutin, suberine, and wax biosynthesis, carbon fixation in photosynthetic organisms, and the MAPK signaling pathway (Figure 1E,F). In the photosynthesis pathway, 18 DEGs were annotated (1 upregulated and 17 downregulated) (Figure 1B, Supplementary Figure S2A). In the glyoxylate and dicarboxylate metabolism pathway, 19 DEGs were annotated (all downregulated). In the nitrogen metabolism pathway, 10 DEGs were annotated (1 upregulated and 9 downregulated). In the cutin, suberine, and wax biosynthesis pathway, 9 DEGs were annotated (5 upregulated and 4 downregulated). In the carbon fixation pathway, 13 DEGs were annotated (all downregulated) (Supplementary Figure S2A). In the MAPK signaling pathway, 21 DEGs were annotated (16 upregulated and 5 downregulated). Overall, GO and KEGG enrichment analyses indicated that *Wolbachia* infection had the most significant negative impact on photosynthesis in cotton leaves. Compared to Tt-UI, important genes involved in photosynthesis were significantly downregulated in Tt-I. Ten significantly differentially expressed genes related to photosynthesis identified from transcriptome sequencing were validated using qRT-PCR. The relative expression levels of these genes were consistent with the sequencing results trends (Supplementary Table S2 and Figure S3).

### 2.2. Gene Co-Expression Network Analysis

**Figure 2:**
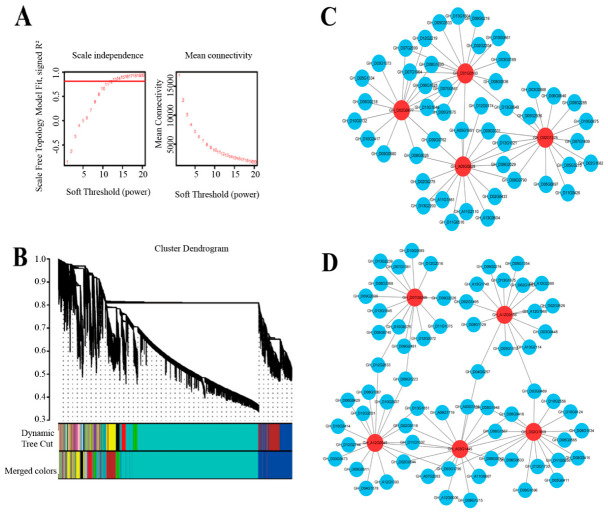
WGCNA gene co-expression network. (**A**) Selection of the soft-threshold value; (**B**) hierarchical clustering dendrogram and module division; (**C**) the photosynthesis core genes in the Red module; and (**D**) carbon fixation core genes of photosynthetic organisms in the Green module.

Weighted gene co-expression network analysis (WGCNA) of RNA-seq data from Tt-I and Tt-UI cotton leaves identified modules linked to photosynthesis. After filtering out genes with low variability from the expression matrix, 26,955 genes were selected for WGCNA analysis. When the optimal soft threshold was set to β = 12, the scale-free topology index (R^2^) exceeded 0.8, and the mean connectivity approached 0 (Figure 2A), indicating that this soft threshold was suitable for constructing a scale-free network. Clustering analysis based on gene expression levels grouped highly co-expressed genes into the same module (Figure 2B). A total of 13 gene co-expression modules were identified, with a significant variation in the number of genes per module. The Turquoise module contained the most genes (17,168), while the Gray module contained the fewest (56). Modules were filtered using Pearson’s correlation coefficient (R > 0.5) and significance level (*p* < 0.05) (Supplementary Figure S4). Notably, the Red (R = 0.95, *p* = 2 × 10^−6^) and Green (R = 0.83, *p* = 0.001) modules showed significant positive correlations.

Cytoscape software (version 3.6.1) was used to visualize the gene co-expression networks. Highly connected genes within each module were identified and designated as core genes. Functional annotation of these genes revealed that the Red module contained four genes playing crucial roles in photosynthesis. These genes ranked within the top 10% for connectivity in the module and included two photosystem II proteins (GH_D01G0160 and GH_D02G1276), one photosystem I protein (GH_D03G0877), and one iron–sulfur cluster domain protein (GH_A05G0828) (Figure 2C). In the Green module, five genes related to the photosynthetic carbon fixation process were identified (Figure 2D), encoding enzymes crucial for the carbon fixation process (Supplementary Table S3).

### 2.3. Effect of Wolbachia on Cotton Amino Acid Levels

**Figure 3:**
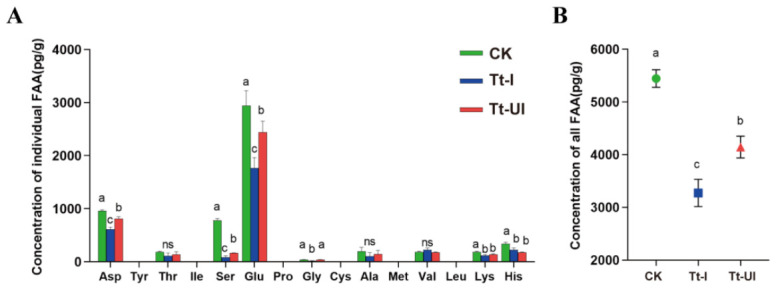
Concentration of free amino acid (FAA) in cotton leaves fed by *Wolbachia*-infected (Tt-I) and uninfected (Tt-UI); CK: cotton leaves not fed on by spider mites as a negative control. (**A**) Free amino acid concentration (FAA); (**B**) total amino acid concentration. Different letters above the column indicate significant differences (*p* < 0.05), ns: not significant.

The free amino acid and total amino acid contents were measured in Tt-I and Tt-UI cotton leaves. The concentrations of aspartic acid (Asp), serine (Ser), and glutamic acid (Glu) were significantly lower in Tt-I leaves compared to both the Tt-UI and control (CK) groups, indicating a *Wolbachia*-associated reduction in these key amino acids (Asp: F_2,6_ = 74.843, *p* < 0.001; Ser: F_2,6_ = 641.760, *p* < 0.001; Glu: F_2,6_ = 19.963, *p* = 0.002) (Figure 3A). Additionally, the total amino acid concentration in Tt-I leaves was significantly reduced (F_2,6_ = 106.371, *p* < 0.001) (Figure 3B). The results demonstrate a distinct *Wolbachia*-associated net reduction in amino acids in cotton leaves.

### 2.4. Effect of Wolbachia on Leaf Chlorophyll Content

**Figure 4:**
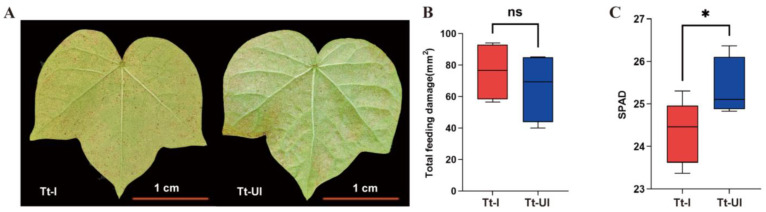
Cotton leaves fed with *Wolbachia*-infected (Tt-I) and uninfected (Tt-UI) spider mites. (**A**) Injury phenotype of cotton leaves; (**B**) damaged leaf area of cotton leaves; (**C**) chlorophyll content value by SPAD (Soil and Plant Analyzer Development). The horizontal lines in the box represent the median, and the thin lines represent the 10th to 90th percentiles. ns: not significant. *: *p* < 0.05. bar = 1 cm. Data are presented as mean ± standard deviation (SD). Significant differences between groups were determined using an independent samples *t*-test (*p* < 0.05).

After one week of feeding by *Wolbachia*-infected (Tt-I) and uninfected (Tt-UI) *T. turkestani*, distinct damage patterns were observed on cotton leaves. Tt-I-infested leaves showed prominent necrotic spots, whereas Tt-UI-infested leaves exhibited chlorotic spots (Figure 4A). The damage area was 75.93 ± 9.327 mm^2^ for Tt-I leaves and 66.04 ± 11.16 mm^2^ for Tt-UI leaves, with no significant difference between them (*p* = 0.52, t = 0.6801, df = 6) (Figure 4B). However, SPAD measurements revealed a significant reduction in the chlorophyll content in Tt-I leaves (SPAD value = 24.35 ± 0.26) compared to Tt-UI leaves (SPAD value = 25.37 ± 0.23) (*p* = 0.011) (Figure 4C). This indicated that *Wolbachia* infection decreased chlorophyll content in cotton leaves, impairing photosynthesis.

### 2.5. Effects of Wolbachia on Leaf ROS Levels

**Figure 5:**
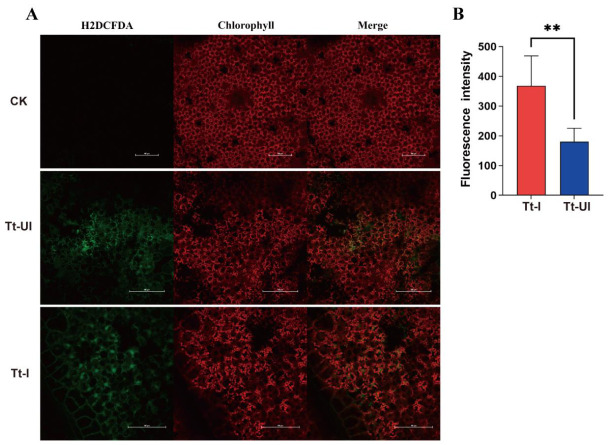
Laser confocal fluorescence imaging of cotton leaves after feeding by spider mites. (**A**) Tt-I: leaves infected with endosymbiotic bacteria, Tt-UI: leaves not infected with endosymbiotic bacteria, CK: cotton leaves not fed on by spider mites; (**B**) fluorescent signal intensity (*n* = 5). Red is the spontaneous fluorescence of chlorophyll, and green is the fluorescence resulting from the oxidation of the fluorescent dye DCFH-DA. Bars = 100 µm. **: *p* < 0.01.

Reactive oxygen species (ROS) are highly reactive molecules, including superoxide anions, hydrogen peroxide, and hydroxyl radicals. ROS levels indicate the extent of oxidative stress-induced cellular damage. To examine whether *Wolbachia*-infected spider mites impaired photosynthesis and simultaneously induced oxidative stress, intracellular ROS levels were quantified using the fluorescent probe DCFH-DA (Solarbio, Beijing, China), where green fluorescence intensity is proportional to ROS levels (Figure 5A). The results showed that Tt-I leaves (367.77 ± 45.05) exhibited twice the fluorescence intensity compared to Tt-UI leaves (180.17 ± 20.27), indicating significantly higher ROS accumulation in Tt-I leaves (*p* < 0.01) (Figure 5B).

## 3. Discussion

This study provides evidence that the endosymbiont *Wolbachia* in the spider mite *T. turkestani* disrupts cotton photosynthesis through transcriptional reprogramming and induction of oxidative stress. Our findings significantly advance the understanding of plant–herbivore–microbe interactions underlying a symbiont-mediated alteration in plant primary metabolism [20].

### 3.1. Wolbachia-Induced Transcriptional Suppression of Photosynthesis

Our transcriptomic analysis reveals the profound downregulation of photosynthetic genes in cotton leaves infested by *Wolbachia*-infected mites. The observed reduction in chlorophyll content aligns with previous studies demonstrating that herbivory can lead to chlorosis and impaired photosynthesis [11,21]. However, our study reveals a more targeted mechanism: *Wolbachia* infection suppresses the expression of critical photosynthetic components, such as psbW encoding photosystem II proteins and ferredoxin (PETF). The significant downregulation of these hub genes, identified via WGCNA, suggests a direct assault on the photosynthetic electron transport chain, potentially disrupting light energy conversion and carbon assimilation efficiency [22]. This transcriptional suppression extends to the carbon fixation pathway, indicating a comprehensive inhibition of the photosynthetic apparatus at the genetic level, which likely underlies the observed physiological decline [23].

### 3.2. The Interplay Between Oxidative Stress and Photosynthetic Inhibition

Beyond transcriptional suppression, our data highlight oxidative stress as a key component of *Wolbachia*-induced phytotoxicity [24]. The elevated ROS levels in Tt-I leaves are not merely a symptom of damage but likely act as a mechanistic link between mite herbivory and photosynthetic decline. Excessive ROS can cause peroxidation of thylakoid membranes and degrade key photosynthetic proteins, thereby exacerbating the damage initiated by the transcriptional downregulation of photosystem components [17,25]. Furthermore, ROS are known to inhibit the activity of carbon fixation enzymes like Rubisco [18], creating a vicious cycle that further compromises plant vitality [26]. This synergy between transcriptional repression and ROS-mediated oxidative damage exemplifies a sophisticated strategy whereby a microbial symbiont amplifies the impact of its host on the plant [27].

### 3.3. Amino Acid Dynamics: A Wolbachia-Associated Nutritional Shift

Our physiological measurements revealed a net reduction in specific free amino acids (e.g., Asp, Ser, and Glu) in cotton leaves infested with *Wolbachia*-infected spider mites (Figure 4). While this finding is consistent with an increased nutrient demand, the exact fate of these depleted amino acids cannot be definitively determined from our current data [28]. This phenomenon could be attributed to several non-mutually exclusive mechanisms: (1) an increased consumption by the *Wolbachia*-infected spider mites themselves, potentially due to an altered metabolic state or enhanced feeding rate induced by the endosymbiont; (2) direct assimilation and utilization by the *Wolbachia* bacteria for its own replication and metabolism within the mite host; or (3) a combination of both processes. Future studies employing techniques such as isotope labeling to trace the flux of nitrogenous compounds from the plant to the mite and its endosymbiont will be crucial to elucidate the primary sink for these amino acids. Nevertheless, the observed *Wolbachia*-dependent shift in leaf amino acid profiles represents a significant alteration in the host plant’s physiological state [29].

This shift potentially creates a more favorable nutritional environment for the mites and their symbionts, aligning with the endosymbionts manipulating host plant physiology for the benefit of their insect hosts [30]. While the “green island” phenomenon induced by other symbionts delays leaf senescence to maintain a feeding site [7], our study reveals an alternative strategy where *Wolbachia* appears to actively manipulate primary metabolic processes (photosynthesis and nitrogen metabolism) to the detriment of the plant, underscoring the diverse evolutionary paths of these interactions.

**Figure 6:**
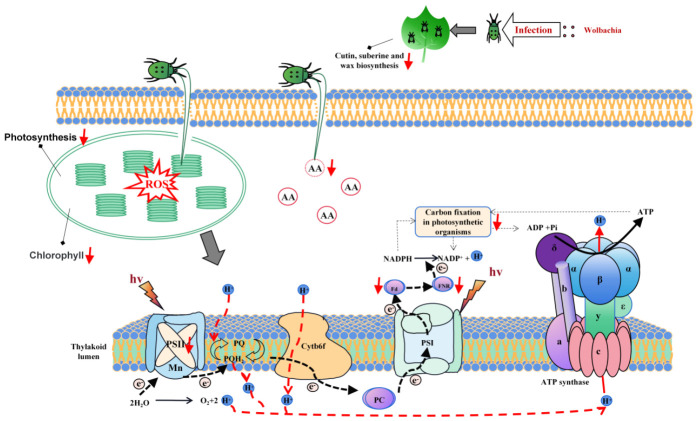
A model of *Wolbachia*-mediated disruption of photosynthesis in cotton by *T. turkestani*. AA: amino acid; ROS: reactive oxygen species. Red arrows indicate downregulation.

In summary, our integrated analysis demonstrates that *Wolbachia* infection in *T. turkestani* disrupts cotton photosynthesis via a dual mechanism of transcriptional repression and ROS-mediated oxidative stress (Figure 6). These findings provide novel mechanistic insights into the role of endosymbionts in modulating plant–herbivore interactions. More importantly, this study suggests that targeting *Wolbachia* could represent a novel and sustainable strategy for reducing mite damage in cotton crops. Future research should focus on exploring specific *Wolbachia*-inhibition techniques and breeding cotton varieties resistant to this symbiotic manipulation, thereby contributing to reduced pesticide reliance in agriculture. Understanding such complex multitrophic interactions is crucial for developing innovative and sustainable pest management strategies.

When *T. turkestani* feeds on cotton leaves, the endosymbiont *Wolbachia* significantly suppresses photosynthetic and carbon fixation pathways. This leads to marked reductions in chlorophyll and amino acid content in the leaves. Concurrently, mite feeding triggers a sharp increase in reactive oxygen species (ROS), inducing oxidative stress that further compromises the structural and functional integrity of the photosynthetic apparatus. Key components of PSII, such as ferredoxin (Fd), and enzymes FNR were affected, and important genes involved in photosynthesis, such as psbW, PETF, FD3, and PSBY, were downregulated.

## 4. Materials and Methods

### 4.1. Plant Materials and Growth Conditions

Cotton (Gossypium hirsutum, cultivar ‘Zhongmian 36’) seeds were sown in pots (20 cm in diameter) filled with a sterilized substrate mixture of peat, vermiculite, and perlite (*v*:*v*:*v* = 3:1:1). Plants were grown in a controlled-environment growth chamber under a 16 h/8 h light/dark cycle at 25 ± 2 °C. A compound fertilizer (N-P-K: 15-15-15) was applied weekly at a concentration of 1 g/L through irrigation water, starting from the two-true-leaf stage.

### 4.2. Mite Colony

*T. turkestani* mites were collected in June 2023 from the experimental field of the College of Agriculture at Shihezi University (44°19′ N, 86°03′ E). The mites were reared on cotton leaves in a light-controlled incubator set at 25 °C with a 16L/8D photoperiod and 60% relative humidity, without exposure to any pesticides.

### 4.3. Detection of Wolbachia Infection in T. turkestani Mites

Total DNA was extracted from *T. turkestani* mites following the method described by Yang Kun et al. [31]. Specific primers for the *Wolbachia* wsp gene were designed (Supplementary Table S2). PCR reactions (25 μL total volume) included 2.0 μL DNA template, 14.8 μL ddH_2_O, 2.5 μL of 10× buffer, 2.0 μL of dNTPs, 1.5 μL of MgCl_2_, 0.2 μL of Taq polymerase, and 0.5 μL each of forward and reverse primers (20 mmol/L). The PCR conditions were as follows: initial denaturation at 94 °C for 2 min, followed by 35 cycles of denaturation at 94 °C for 30 s, annealing at 55 °C for 45 s, and extension at 72 °C for 45 s, with a final extension at 72 °C for 5 min. PCR products (15–20 μL) were subjected to 1% agarose gel electrophoresis for detection.

### 4.4. Screening of Different Infected Strains of T. turkestani

A parthenogenetic backcrossing method was used to select different *Wolbachia* infection strains of *T. turkestani*. Fresh whole bean leaves were placed in Petri dishes (9 cm in diameter) lined with sponges. The leaves were divided into 3–5 approximately equal compartments using moist cotton strips based on their size. One unmated deutonymph female mite was isolated into each compartment for parthenogenetic reproduction of males. Once the eggs developed into adult males, they were backcrossed with the mother for two days before transferring the female to a new compartment for oviposition. After seven days, the mother mite was tested for *Wolbachia* infection using PCR. The offspring from *Wolbachia*-infected mothers were subjected to the above process for four to five generations. If 50 female adult offspring tested positive for *Wolbachia* infection, the strain was considered suitable for experiments. To obtain *Wolbachia*-free strains, bean leaves were soaked in 0.1% tetracycline for 24 h and used to feed newly hatched *T. turkestani* larvae. Fifty adult females were tested per generation using PCR. Strains were deemed *Wolbachia*-free if no infection was detected. To ensure the complete absence of *Wolbachia*, the putatively uninfected strain was maintained for 10 consecutive generations without tetracycline treatment. At the end of the 10th generation, the absence of *Wolbachia* was re-confirmed by PCR on 50 adult females before they were used in the experiments [32].

### 4.5. Infestation Process of Host Cotton by T. turkestani

The selected *T. turkestani* strains were acclimatized on cotton plants. For each strain, five 21-day-old cotton plants were selected, and three leaves per plant were chosen. Mites aged 3 ± 1 days were transferred onto the experimental cotton leaves, with 30 mites per leaf. The infection lasted for seven days. To prevent mite escape, moistened cotton was placed around the petioles. The number of adult mites was monitored daily, and any escaped or dead mites were promptly replaced. After seven days of infestation, cotton leaves were collected. Before sampling, a fine brush was used to gently remove the spider mites from the leaves, ensuring no mite mouthparts or other body parts remained on the leaf surface. Leaves were then quickly (within 5 s) cut off using autoclaved scissors. The samples were immediately flash-frozen in liquid nitrogen for 10 min, and then transferred to a −80 °C ultra-low temperature freezer for cryogenic storage, intended for transcriptome sequencing analysis [33]. Three independent replicates were used for each treatment. Leaves infected with *Wolbachia*-infected spider mites were labeled Tt-I_1-3, while leaves infected with *Wolbachia*-free spider mites were labeled as Tt-UI_1-3.

### 4.6. Transcriptome Sequencing of Cotton Leaves from Different Infected Lines

Total RNA was extracted from cotton leaves using TRIzol reagent. Library construction and sequencing were performed by [Novogene Co., Ltd., Beijing, China]. Briefly, sequencing libraries were prepared with the Illumina NEBNext Ultra™ RNA Library Prep Kit and sequenced on an Illumina HiSeq 3000 platform to generate 150 bp paired-end reads. Raw sequence data were processed and quality-controlled. The clean reads were then aligned to the *Gossypium hirsutum* reference genome (http://cotton.zju.edu.cn/download.html) (accessed on 20 May 2024) using HISAT2. Gene expression levels were quantified based on the FPKM method. Differential gene expression analysis between the Tt-I and Tt-UI groups was conducted using the DESeq2 package with a threshold of |log_2_(FoldChange)| > 0 and *p*-value < 0.05. Gene Ontology (GO) and Kyoto Encyclopedia of Genes and Genomes (KEGG) pathway enrichment analyses of differentially expressed genes (DEGs) were performed using the clusterProfiler package.

### 4.7. Transcriptome-Based Analysis of Weighted Gene Co-Expression Networks

To identify co-expressed gene modules associated with the treatment, we performed a weighted gene co-expression network analysis (WGCNA) using the WGCNA R package (version 1.72-1). The analysis was based on the expression matrix of all expressed genes across the six samples. Genes with highly correlated expression patterns were grouped into modules. The detailed parameters for network construction are provided in the Supplementary Methods. The resulting module–trait relationships were examined, and key modules of interest (e.g., those significantly correlated with the *Wolbachia*-infection trait) were selected for downstream functional analysis and identification of hub genes. Visualization of the network was performed using Cytoscape v3.6.1.

### 4.8. Determination of Free Amino Acid Content in Cotton Leaves

Free amino acid content in cotton leaves was analyzed according to the method described by Kang et al. [34] with minor modifications. Briefly, 250 mg of cotton leaf tissue was homogenized and extracted using 5 mL of 2% trichloroacetic acid. The filtrate was dried and subsequently dissolved in 0.02 M HCl, followed by centrifugation at 11,800 rpm for 15 min. Free amino acids in the supernatant were then quantified using an automatic amino acid analyzer (Hitachi L-8900, Tokyo, Japan). The quantification was based on the external standard method, employing a known concentration of amino acid mixed standard (Sigma-Aldrich, St. Louis, MO, USA) to establish a calibration curve. The standard curve consisted of six concentration gradients (0.1–50 μmol/L), with linear correlation coefficients (R^2^) all greater than 0.995, ensuring the accuracy and linear range of the quantification. Instrument calibration and quality control were performed using standards prior to the analysis of each batch of samples. Untreated healthy cotton leaves, serving as the control group, are denoted as CK.

### 4.9. Determination of Cotton Leaf Area Damaged and Chlorophyll Content

Seven days after mite infestation, all mites and eggs were removed from the cotton leaves. The leaves were gently flattened using a thin glass plate and photographed under a microscope to assess the damaged area caused by mite feeding. The feeding area was estimated using the software “Compu Eye, Leaf & Symptom Area” (http://www.ehabsoft.com/CompuEye/LeafSArea) (accessed on 15 October 2025) [35]. For the assessment of the feeding damage area, each treatment was conducted in three replicates (Tt-I_1-3 and Tt-UI_1-3), with four leaves measured per replicate.

Chlorophyll content was measured using a SPAD (Soil and Plant Analyzer Development), a portable chlorophyll meter (model SPAD-502, Konica Minolta, Tokyo, Japan). For the Tt-UI and Tt-I strains, eight cotton leaves were selected from each strain. Due to positional variation in chlorophyll content, SPAD values were measured at three positions on each leaf (tip, middle, and base), and the average value was calculated. During the measurement, care was taken to avoid damaged areas to ensure data accuracy. Data are presented as mean ± standard deviation (SD). Significant differences between groups were determined using an independent samples *t*-test (*p* < 0.05)

### 4.10. ROS Fluorescence in Situ Hybridization Assay

To assess the level and localization of hydrogen peroxide (H_2_O_2_) accumulation in cotton leaves infected by *Wolbachia*-infected *T. turkestani*, the fluorescent probe 2′,7′-dichlorodihydrofluorescein diacetate (H_2_DCF-DA) (AAT Bioquest, Pleasanton, CA, USA), a fluorescent dye for H_2_O_2_, was used to vacuum infiltrate Tt-I and Tt-UI cotton leaves in phosphate-buffer solution (PBS) at pH 7.4. Uninfected cotton leaves were used as a blank control. Leaf sections of 1 cm were cut from the same positions of the three samples using scissors and quickly immersed in a solution containing 10 μmol·L^−1^ H_2_DCF-DA. Vacuum infiltration was conducted using a sealed 100 mL syringe by moving the piston back and forth several times for 10–30 s to allow the H_2_DCF-DA to infiltrate plant tissues. Endogenous plant esterases then cleaved the acetate groups, releasing H_2_DCF, which then reacted with H_2_O_2_ in the presence of endogenous peroxidases to produce the fluorescent compound DCF. After incubation in the dark at room temperature for 10 min, the leaf samples were washed with 10 mmol·L^−1^ Tris-HCl buffer to thoroughly remove any residual H_2_DCF-DA probe that had not entered the leaf cells. Fluorescence was observed using a laser scanning confocal microscope (Nikon, Tokyo, Japan). The fluorescent DCF emitted fluorescence at 510–550 nm, while chloroplasts emitted fluorescence at 650–750 nm [36]. Fluorescence intensity was quantified using ImageJ software (version 1.54k) with six replicates for each treatment.

### 4.11. Statistical Analysis

All data are presented as the mean value ± standard deviation (SD). One-way analysis of variance (ANOVA) of amino acid content was performed using SPSS 20.0 (SPSS Inc., Chicago, IL, USA), and *t*-tests were conducted for leaf damage area, chlorophyll content, and reactive oxygen species (ROS) fluorescence intensity data using GraphPad Prism 7.0 (GraphPad Software Inc., San Diego, CA, USA).

## Data Availability

The original contributions presented in this study are included in the article/Supplementary Material. Further inquiries can be directed to the corresponding authors.

## Supplementary Materials

The following supporting information can be downloaded at: https://www.mdpi.com/article/10.3390/ijms27093746/s1. References [37,38] are cited in the Supplementary Materials.
