# Supplementary material for: Transcriptomic Analysis Reveals Molecular Mechanisms of Wolbachia–Plant Association
Source: Int J Mol Sci. 2026 Apr 23;27(9):3746. doi: 10.3390/ijms27093746 (PMC13164273; doi:10.3390/ijms27093746)
Supplement: Supplementary file 1 [file ijms-27-03746-s001.zip › ijms-3544464-supplementary.pdf]

Supplementary Table S1. Summary and Comparative Statistics of RNA - Seq Sequencing Data Quality Tt-I_1-3 are three replicates of leaves fed with *Wolbachia*-infected spider mites were labeled Tt-I1-3, while leaves fed with *Wolbachia*-free spider mites were labeled as Tt-UI_1-3

**Sample**	**Raw_Reads**	**Clean_Reads**	**Clean_Bases**	**Error_Rate**	**Q20**	**Q30**	**GC_pct**	**Total_Map**
Tt-I _1	47305380	47271448	7.09G	0.01	98.29	95.09	43.59	43797145(92.65%)
Tt-I _2	47853272	47809388	7.17G	0.01	98.47	95.61	43.66	44764897(93.63%)
Tt-I _3	40619308	38016840	5.7G	0.01	98.3	95.19	43.5	35352409(92.99%)
Tt-UI _1	41025864	38214828	5.73G	0.01	98.28	95.13	43.57	35528696(92.97%)
Tt-UI _2	42335284	39832862	5.97G	0.01	98.34	95.3	43.51	36912372(92.67%)
Tt-UI _3	40499776	38448060	5.77G	0.01	98.38	95.37	43.37	35637258(92.69%)

Supplementary Table S2. The primers used in this study

Primer name	Sequences	Destination
GhUBQ7F	GAAGGCATTCCACCTGACCAAC	qRT-PCR
GhUBQ7R	CTTGACCTTCTTCTTCTTGTGCTTG	qRT-PCR
GH_D02G2344-F	CAAAGAAAAGGCCGCTTTACCA	qRT-PCR
GH_D02G2344-R	TTAGGACGAAGCCTGCATCAAT	qRT-PCR
GH_A13G0356-F	CGGAAGGACCTCAAGAGTTTGA	qRT-PCR
GH_A13G0356-R	ATGACGACATCACTTGTTGGGA	qRT-PCR
GH_A13G1667-F	TAGCCTTTTCCTCCATTGCACT	qRT-PCR
GH_A13G1667-R	GCGTTCGTAATCTCTGCTGTTC	qRT-PCR
GH_A01G2050-F	CAAGTACCAGCGTTTCAACCAG	qRT-PCR
GH_A01G2050-R	GCAGCAACAGTGTTAGAGTGTG	qRT-PCR
GH_D11G1743-F	GTGTTGTTTTCTAGGTGTGGCC	qRT-PCR
GH_D11G1743-R	GGGTAGGCTCGAGTATTTACGG	qRT-PCR
GH_A13G2454-F	GGGTTGGCAAGGTCTCTAATCA	qRT-PCR
GH_A13G2454-R	TCGCCCTTAGGACTAGAGTCAA	qRT-PCR
GH_D11G1742-F	GTGTTGTTTTCTAGGTGTGGCC	qRT-PCR
GH_D11G1742-R	GGGTAGGCTCGAGTATTTACGG	qRT-PCR
GH_A08G2604-F	ATGCCACTTCCCAAGCATTAGA	qRT-PCR
GH_A08G2604-R	TCCCATGATAAGGCTTTGCAGT	qRT-PCR
GH_A13G1534-F	GCAGAATCTGGATGCGATGAAC	qRT-PCR
GH_A13G1534-R	CGAATTCGCTTTGCATCTCGAT	qRT-PCR
GH_A09G0049-F	AGTGAGACCCCCACAAGATCTA	qRT-PCR
GH_A09G0049-R	GAAGAAAGGAGCCAAGGGAAGA	qRT-PCR
WSP/F236	GACAGTTTAACAGCATTTTCAGGA	PCR
WSP/R44	GTTTGATTTCTGGAGTTACATCAT	PCR

Supplementary Table S3. Functional annotation of the core genes in the target module

module	Hub gene_ID	Connectivity	homologous gene ID in A.thaliana	gene function
Red	GH_D01G0160	117.7517449	AT1G03600	Photosystem II repair protein PSB27-H1
	GH_D03G0877	145.1684543	AT4G01830	Photosystem I psaA/psaB protein
	GH_D02G1276	161.7288241	AT3G50820	Photosystem II protein
	GH_A05G0828	162.1450069	AT1G60950	2Fe-2S iron-sulfur cluster binding domain
Green	GH_A12G0156	13.59281493	AT5G64380	Fructose-1,6-bisphosphatase
	GH_D07G0269	14.86792067	AT5G25880	NADP-dependent malic enzyme
	GH_A12G0545	32.86766761	AT1G53310	Phosphoenolpyruvate carboxylase
	GH_D02G1668	239.4720939	AT1G13440	Glyceraldehyde-3-phosphate dehydrogenase
	GH_A03G1445	295.5611387	AT2G01290	Probable ribose-5-phosphate isomerase

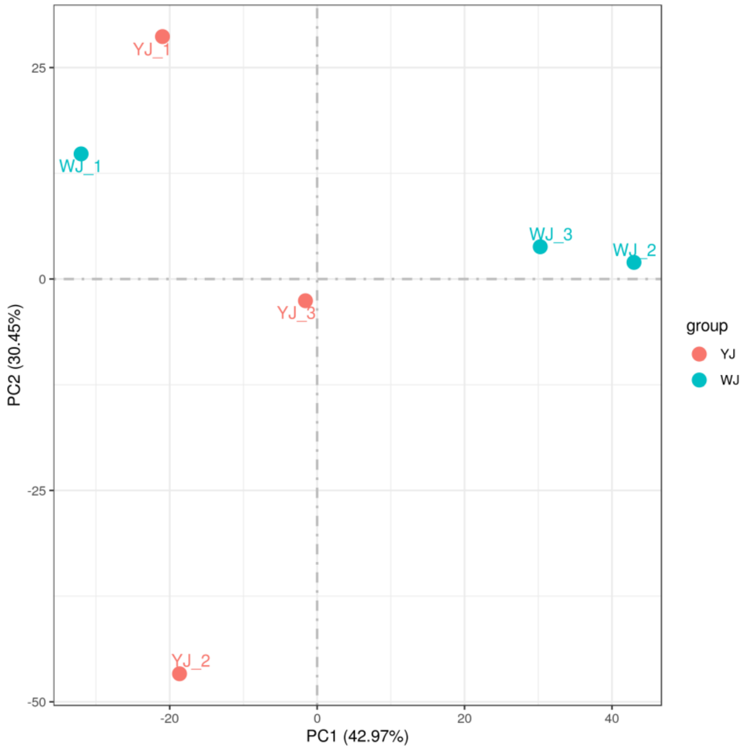

Supplementary Figure S1. Principal Component Analysis

YJ represents Tt-I_1-3 replicates and WJ represents Tt-UI_1-3 replicates of cotton leaves.

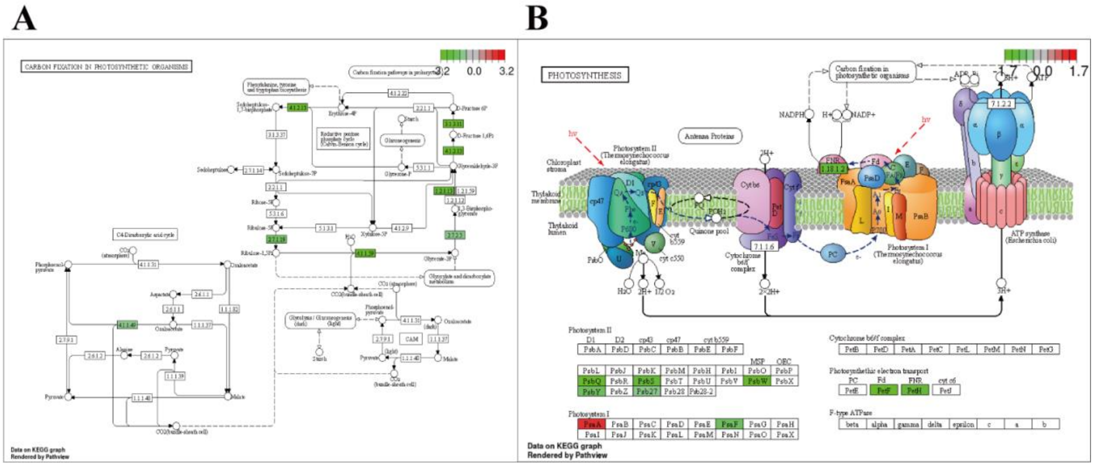

Supplementary Figure S2. KEGG Pathway

(A) DEGs located in photosynthesis (ko00195); (B) DEGs mapped to carbon fixation in photosynthesis (ko00710). Green boxes indicate significantly downregulated genes, while red boxes indicate significantly upregulated genes.

RT-qPCR analysis

cDNA was synthesized from 3 μg of total RNA using MMLV reverse transcriptase (TaKaRa, Dalian, China). RT-qPCR analysis was performed using SYBR Green (Roche, Rotkreuz, Switzerland) on a LightCycler 480II system (Roche). All primers used in the RT-qPCR were designed using Primer3 Plus, as detailed in Supplementary Table S3. The cotton UBQ7 gene (DQ116441.1) was used as the reference gene to normalize variance among samples. The PCR conditions included an initial denaturation at 95°C for 5 min, followed by 40 cycles of denaturation at 95°C for 15 s, annealing at 60°C for 15 s, and extension at 72°C for 20 s. Each reaction was performed in three technical replicates and three independent biological replicates. Relative quantification of target gene expression was calculated using the $2^{-\Delta\Delta CT}$ method according to Livak[1].

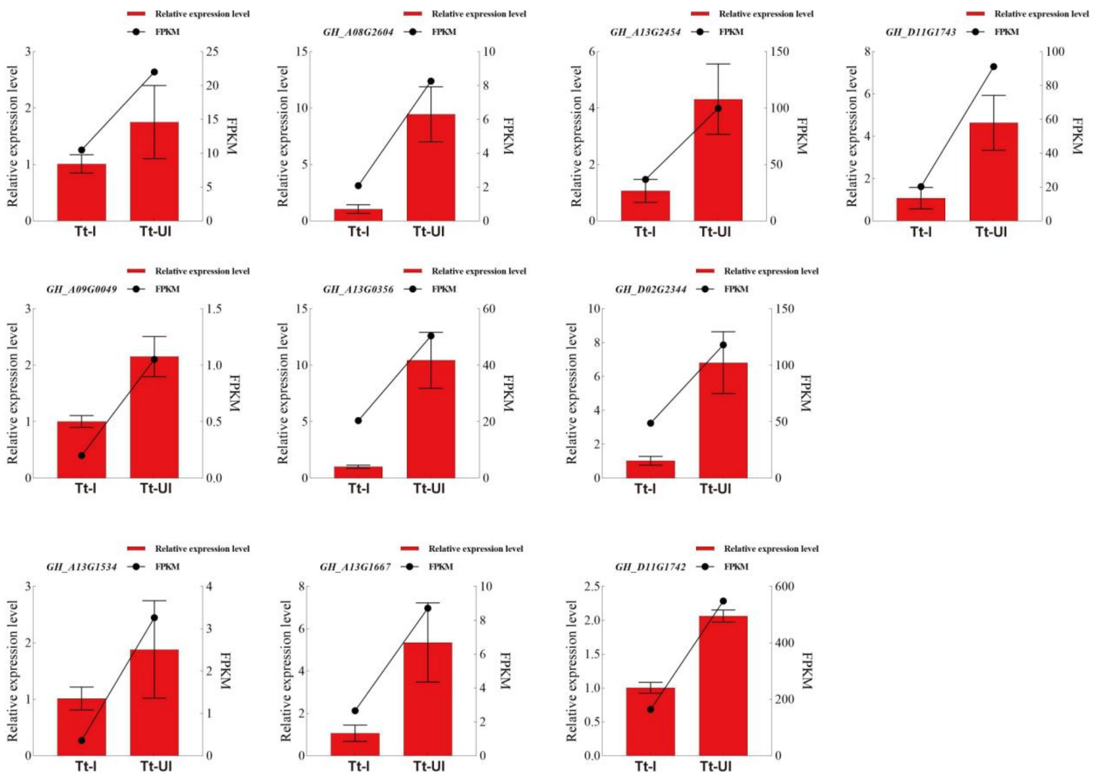

Supplementary Figure S3. Expression of photosynthesis - related genes in Tt-I and Tt-UI cotton leaves detected by qRT-PCR.

[1]Livak, K.J.; Schmittgen, T.D. Analysis of Relative Gene Expression Data Using Real-Time Quantitative PCR and the $2^{-\Delta\Delta CT}$ Method. Methods **2001**, *25*, 402–408, doi:10.1006/meth.2001.1262.

Transcriptome-based analysis of weighted gene co-expression networks

WGCNA Network Construction Parameters: A weighted gene co-expression network was conducted using the WGCNA package (version 1.72-1) in R[2]. The analysis was based on a gene expression matrix comprising six samples: three biological replicates of Tt-UI and three biological replicates of Tt-I. The pickSoftThreshold function was used to calculate the appropriate weighting coefficient β, with the criterion that the square of the correlation coefficient should be approximately 0.8 while maintaining a certain level of gene connectivity. When the optimal soft threshold was set to β=12, the scale-free topology index (R^2) exceeded 0.8, and the mean connectivity approached 0, indicating that this soft threshold was suitable for constructing a scale-free network. Clustering analysis based on gene expression levels grouped highly co-expressed genes into the same module. Visualization of the network was performed using Cytoscape v3.6.1.

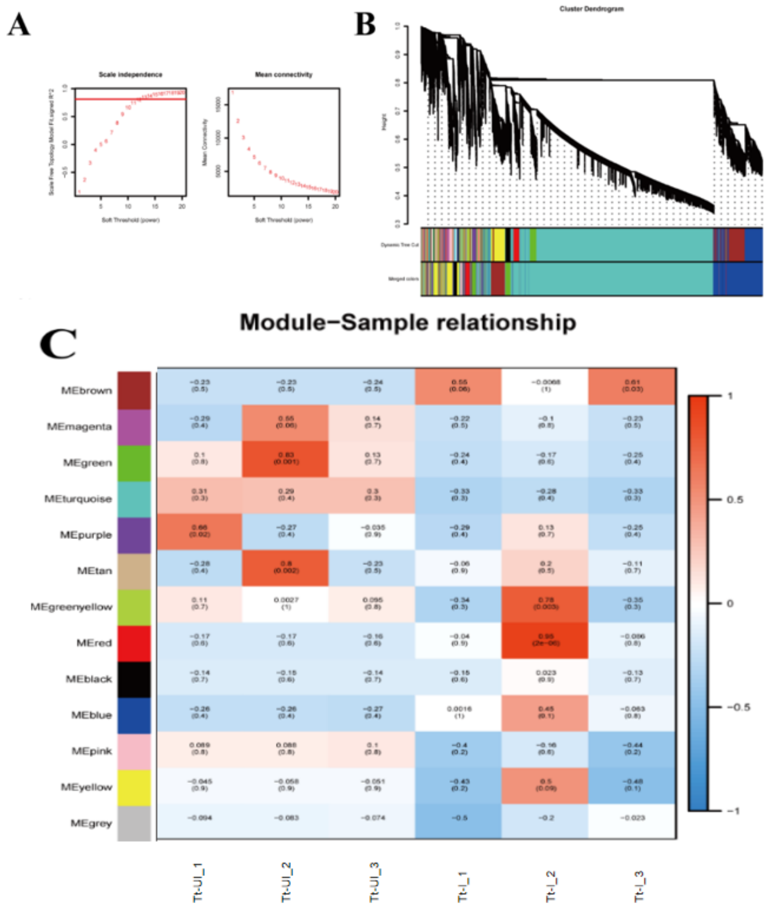

Supplementary Figure S4. Module-sample relationship heatmap in WGCNA co-expression network.

The heatmap displays the Pearson's correlation coefficients between each gene co-expression module (rows) and individual RNA-seq samples (columns). The six columns represent the three biological replicates per treatment (Tt-UI_1, Tt-UI_2, Tt-UI_3 and Tt-I_1, Tt-I_2, Tt-I_3).

[2]Langfelder, P., & Horvath, S. (2008). WGCNA: an R package for weighted correlation network analysis. BMC Bioinformatics, 9, 559. https://doi.org/10.1186/1471-2105-9-559
